# Genetic polyploid phasing from low-depth progeny samples

**DOI:** 10.1016/j.isci.2022.104461

**Published:** 2022-05-25

**Authors:** Sven Schrinner, Rebecca Serra Mari, Richard Finkers, Paul Arens, Björn Usadel, Tobias Marschall, Gunnar W. Klau

**Affiliations:** 1 Algorithmic Bioinformatics, Heinrich Heine University Düsseldorf, Düsseldorf, Germany; 2 Institute for Medical Biometry and Bioinformatics, Medical Faculty, Heinrich Heine University Düsseldorf, Düsseldorf, Germany; 3 Plant Breeding, Wageningen University & Research, Wageningen, the Netherlands; 4 Cluster of Excellence on Plant Sciences (CEPLAS), Heinrich Heine University Düsseldorf, Düsseldorf, Germany; 5 Forschungszentrum Jülich, Institute of Bio and Geosciences, Bioinformatics (IBG-4), Jülich, Germany; 6 Bioeconomy Science Center, c/o Forschungszentrum, Jülich, Germany; 7 Biological Data Science, Heinrich Heine University Düsseldorf, Düsseldorf, Germany; 8 Gennovation B.V., Agro Business Park 10, 6708 PW, Wageningen, The Netherlands

**Keywords:** Bioinformatics, Genomics, Sequence analysis

## Abstract

An important challenge in genome assembly is haplotype phasing, that is, to reconstruct the different haplotype sequences of an individual genome. Phasing becomes considerably more difficult with increasing ploidy, which makes polyploid phasing a notoriously hard computational problem. We present a novel genetic phasing method for plant breeding with the aim to phase two deep-sequenced parental samples with the help of a large number of progeny samples sequenced at low depth. The key ideas underlying our approach are to (i) integrate the individually weak Mendelian progeny signals with a Bayesian log-likelihood model, (ii) cluster alleles according to their likelihood of co-occurrence, and (iii) assign them to haplotypes via an interval scheduling approach. We show on two deep-sequenced parental and 193 low-depth progeny potato samples that our approach computes high-quality sparse phasings and that it scales to whole genomes.

## Highlights

• Allows phasing of autopolyploid species through genetic information of progenies
• High number of low-depth progeny samples yields significant markers for phasing
• Informative variant types (simplex-nulliplex) phasable with high confidence
• Continuity not limited by read connectivity, but rather by the recombination rate

## Graphical abstract

**Figure:**
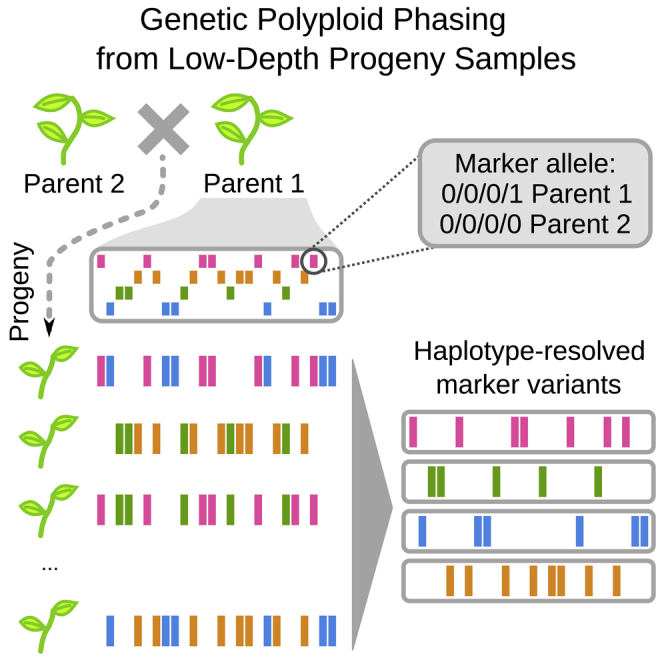

## Introduction

DNA of higher organisms is organized in sets of homologous chromosomes or haplotypes. The cardinality *k* of these sets is the *ploidy* and a general characteristic of an organism. Humans, for example, are diploid (*k* = 2). In contrast, many plant species are polyploid, like the tetraploid potato (*Solanum tuberosum*, *k* = 4) or the hexaploid chrysanthemum (*Chrysanthemum morifolium Ramat*, *k* = 6). An important challenge in genome assembly is haplotype phasing, that is, to reconstruct the *k* different haplotypes of an individual genome. This enables to understand evolutionary events at higher resolution and makes advanced breeding strategies possible. Yet, identifying which alleles co-occur on each of the homologous copies becomes considerably more difficult with increasing ploidy and polyploid phasing is known to be a notoriously hard computational problem.

The predominant method in plant genomics is read-based phasing. Because of limited read length and high similarity of haplotypes, current *de novo* assembly methods are unfortunately unable to produce larger phased blocks, even when using HiFi reads. For this reason, the reads of a sample are usually first aligned to a reference genome which reveals all heterozygous positions where the reads differ from each other. Co-occurring alleles on overlapping reads can then be used to partition the reads into clusters that correspond to partial haplotypes. Current state-of-the-art tools include Flopp (Shaw and Yu, 2022), HPoP-G (Xie et al., 2016), nPhase (Abou Saada et al., 2021), Ranbow (Moeinzadeh et al., 2020), and WhatsHap Polyphase (Schrinner et al., 2020). A common problem for read-based polyploid phasers is switch errors, i.e. variant sites in the phasing, where two or more haplotypes are linked in a wrong way. In addition, so-called *collapsed regions*, where a subset of haplotypes is identical on longer regions of the genome (Schrinner et al., 2020), are hard to resolve by reads alone.

Genetic phasing links alleles on haplotypes by using samples from the same pedigree and inferring the transmission of alleles using Mendelian inheritance rules. This has been conducted for diploid data (Abecasis et al., 2002; Williams et al., 2010), also by extending read-based phasing to small pedigrees (Garg et al., 2016). For polyploid data, Tri-Poly (Motazedi et al., 2018) uses read data from parent-child trios to estimate haplotypes. A follow-up approach PopPoly (Motazedi et al., 2019) is able to include multiple progeny samples to increase phasing accuracy.

As the high number of possible haplotype combinations in auto-polyploid progeny renders the classical trio-phasing less effective than for human data, we consider the special case of two hi-depth parental samples and a large number of low-depth progeny samples, which in case of plants are relatively easy to generate on demand. Although the low coverage does not allow to directly phase the progeny samples, our novel approach utilizes their genetic information to infer the parent haplotypes without depending on good read connectivity.

The core idea is to determine which alleles of the parent samples lie on the same haplotype by combining informative parental variant pairs and their allele depths in the progeny samples. We integrate these individually weak signals with a Bayesian log-likelihood model that incorporates Mendel’s law of segregation. In the next step, we cluster alleles according to their likelihood of co-occurrence on a parental haplotype. Finally, we compute a maximum conflict-free assignment of marker alleles to haplotypes by an interval scheduling model.

We demonstrate the feasibility of our approach on data of the tetraploid *Solanum tuberosum*. The study consists of two deep-sequenced parental samples and 193 progeny samples with ∼6x short-read coverage, that is, ∼1.5x per haplotype. We show on three selected regions where we could read off a ground-truth phasing from a HiFi read assembly that our approach produces high-quality sparse haplotype skeletons with Hamming error rates less than 3% over regions containing several thousand variants. Although still many unphased variants in these regions exist, these skeletons will prove useful as anchors in a combination with read-based approaches. We also show that our approach scales to whole genomes by phasing each chromosome of the potato genome in less than 40 CPU hours. We demonstrate that the resulting haplotype blocks are significantly longer than what pure read-based approaches could achieve.

## Results

### WhatsHap polyphase genetic (WH-PPG)

Our key finding is the developed method that phases polyploid samples via the use of a large offspring panel with low individual sequencing depth. As a scenario, we have genotype data for two parental input samples *s*' and *s*'' and allele counts for a set of progeny samples *s*_1_, …,*s*_*p*_. The goal is to phase one of the parent samples, say *s*', based on genetic information offered by the offspring panel.

The type of variants, i.e. the number of distinct alleles and the parental genotypes, play an important role. We refer to biallelic variants as variants for which only two different alleles exist among all samples. We call the more frequent allele among *s*' and *s*'' the *majority* allele and the other one the *minority* allele. Let all biallelic, heterozygous variants for *s*' be numbered from one to *m*. In this setting, the genotype of sample *s* for a single variant *i* can be expressed as an integer number $G_{s}^{i}\in \left\{0,\ldots ,k\right\}$ counting the occurrences of the minority allele, where *k* is the ploidy. If $G_{s^{\prime }}^{i}=1$ and $G_{s^{\text{″}}}^{i}=0$, we call *i* a *simplex-nulliplex* variant.

**Figure 1:**
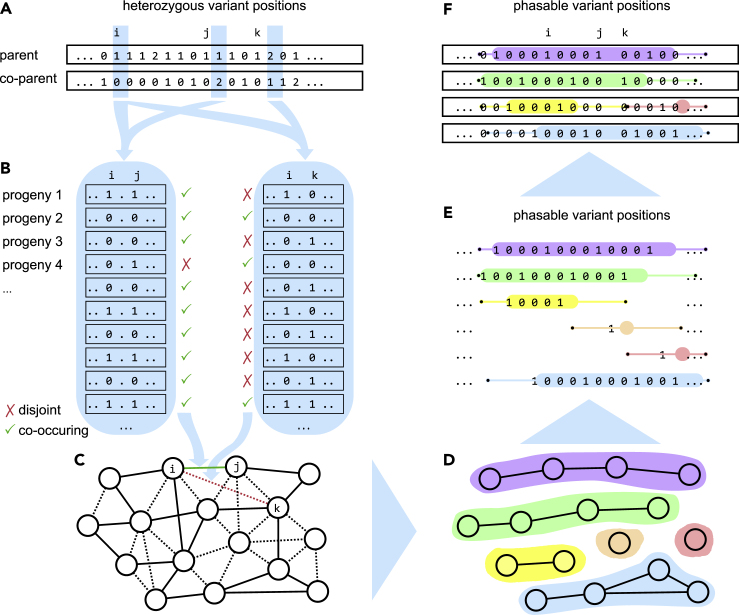
Method overview (A) Genotypes of both parent samples are scanned for informative variants. For illustration purposes, we focus on simplex-nulliplex variant pairs in this overview. (B) Based on progeny allele depths (here we just show progeny genotypes for the sake of simplicity) every variant pair is either classified as having alt-alleles (markers) on the same haplotype (green check mark) or not (red cross). (C) We compute a Bayesian log-likelihood score for each edge in a complete graph where the nodes are alleles (only edges with non-zero score are drawn). (D) Clustering determines groups of markers belonging to the same haplotype. (E and F) (E) Based on the positions of these clusters in the variant space we use an interval scheduling approach to select a maximum conflict-free subset that corresponds to *k* haplotypes (here *k* = 4) (F).

The full method consists of a variety of algorithmic steps which are visualized in Figure 1. We start by identifying variant types that are most informative for Mendelian inference rules – usually simplex-nulliplex variants, because they contain a unique and easy-to-trace allele. Each pair *i*,*j* of picked variants is scored by a Bayesian model, where we decide for each progeny sample *s* whether the allele depths from the input are better explained by placing the two alleles of *i* and *j* in *s*' on the same or on different haplotypes. This results in a graph with one vertex per variant and log-likelihood scored edge weights. Using a clustering model we obtain clusters of alleles that should be placed on the same haplotype for the phased parent sample *s*'. As the number of clusters does not necessarily match the ploidy, we apply an interval scheduling model to find an assignment of clusters to haplotypes that maximizes the number of assigned alleles and avoids conflicts with respect to the Bayesian scoring.

A detailed description for each of the steps is available in the STAR Methods section. In particular, the core idea of statistically infering co-occurences of marker alleles from simplex-nulliplex variants is highlighted in Figure 5. Our algorithm (WH-PPG) is available as part of the widely-used WhatsHap phasing suite.

### WH-PPG produces accurate phasings

We conducted experiments on two parent samples of *S. tuberosum*, named “Altus” and “Colomba” and 193 progeny samples. Each of the samples has been sequenced using Illumina sequencing technology with 250 bp paired-end reads. The average sequencing depth is ∼6x for each progeny sample and more than 300x for each parent sample. All reads have been aligned to the Solyntus V1.1 reference genome (van Lieshout et al., 2020) and variant calling has been performed using GATK (Poplin et al., 2018). In addition, we have a library of HiFi reads for Altus with an average coverage of 24x per haplotype.

In order to evaluate the accuracy of our method, we used the HiFi reads to create four ground truth haplotypes for small stretches of the genome. We computed an assembly graph over these reads using hifiasm v0.13 with standard settings (Cheng et al., 2021), aligned the node sequences to the reference genome and selected three regions on chromosomes 3, 4, and 5 that were continuously covered by four contigs each. Despite their relatively small size of about 300 kb, they were among the largest of their kind, as it proved quite difficult to find long regions with four clearly visible haplotypes based on the assembly alone. We extracted these regions from the VCF files and the HiFi read file.

We ran WH-PPG on all three regions with default settings. In addition, simplex-simplex and duplex-nulliplex variants have been added successively in separate runs. The total number of heterozygous variants and number of bi-allelic ones is also given as comparison in Table 1, Table 2, Table 3.

**Table 1:** Error metrics for WH-PPG on chromosome 3 region

Variant types	Phased	SER (%)	WGR (%)	HR (%)
only simplex-nulliplex	3,934	0.97	3.46	2.89
(50% sampled)	3,581	0.86	3.27	2.55
(25% sampled)	3,164	0.50	6.89	4.2
+ simplex-simplex	3,927	0.95	3.26	2.63
+ duplex-nulliplex	5,943	3.25	20.46	8.88

Regions coordinates are ch03:60,269,000–60,504,000 with 9,549 biallelic and 10,286 total variants. Reported metrics are switch error rate (SER), wrong genotype rate (WGR), Hamming rate (HR), and the total number of phased variants. The exclusive simplex-nulliplex-mode has been repeated with 50 and 25% of parental coverage for genotype calling.

**Table 2:** Error metrics for WH-PPG on chromosome 4 region

Variant types	Phased	SER (%)	WGR (%)	HR (%)
only simplex-nulliplex	3,127	0.26	1.37	0.63
(50% sampled)	2,948	0.47	1.42	0.81
(25% sampled)	2,562	0.51	1.83	1.08
+ simplex-simplex	4,289	1.37	2.19	1.63
+ duplex-nulliplex	4,508	1.57	3.02	2.02

Regions coordinates are ch04:71,586,000–71,947,000 with 12,378 biallelic and 14,500 total variants. Columns and rows have follow the same scheme as Table 1.

**Table 3:** Error metrics for WH-PPG on chromosome 5 region

Variant types	Phased	SER (%)	WGR (%)	HR (%)
only simplex-nulliplex	5,096	0.82	0.90	0.92
(50% sampled)	4,634	0.47	0.73	0.56
(25% sampled)	4,075	0.52	0.69	0.60
+ simplex-simplex	5,332	0.73	1.41	0.95
+ duplex-nulliplex	6,350	0.99	9.78	3.33

Regions coordinates are ch05:56,711,000–57,066,000 with 13,030 biallelic and 15,810 total variants. Columns and rows have follow the same scheme as Table 1.

As error metrics, we use the Hamming rate (HR) and switch error rate (SER). They are defined in the same way as in (Schrinner et al., 2020) and (Shaw and Yu, 2022). The Hamming rate searches for a mapping between predicted and true haplotypes with the lowest fraction of incorrect alleles when comparing the pairs of corresponding haplotypes. The SER is similar, but instead of counting incorrect alleles, it counts how many switches are necessary to transform the predicted haplotypes into the true. As this method can only be applied to positions with matching genotypes, we additionally state the wrong genotype rate (WGR), the fraction of positions in which the predicted genotypes do not match the ground truth genotypes. These might not all necessarily be errors, as the input genotypes used by WH-PPG were created from different data than the ground truth genotypes and might be more susceptible to mapping errors owing to the short reads behind them.

The Hamming rates on chromosome 4 and 5 regions are less than 1% on default settings. This proves the overall correctness of the computed phasing. For the chromosome 3 region this error rate grows to almost 3% which can be explained by the increased number of genotype deviations compared to the other two regions.

Depending on the chromosome WH-PPG is able to phase about 25–41% of the bi-allelic variants. Including simplex-simplex and duplex-nulliplex variants increases the number of phased variants to 36–61% at the cost of tripling the Hamming rate. It can be noted in general that the duplex-nulliplex variants introduce a lot of genotype deviations. This indicates that many of these variants might be mis-classified by the variant caller. All computed phasings consist of one phasing block only.

As the parental coverage was relatively high in the initial runs, we repeated the experiments for the simplex-nulliplex instances, but only used 50 and 25% of the parental reads, respectively. That is, we used GATK to downsample the parental read data to 50 and 25% on chromosomes 3, 4, and 5 and reran WH-PPG on the newly called variants. The number of phased variants decreases consistently with lower coverage, resulting in a total decline of about 20%. The main cause is likely genotype shifts during the variant calling owing to different (and less) read information. Simplex-nulliplex variants in the full dataset shift into another variant much more often than the other way round. The error rates follow no clear pattern throughout the coverage reduction. One would expect them to grow along with the uncertainty of the variant calling like for the chromosome 4 region, but the other two regions rather see lower error rates with lower coverage. Aside from observing this phenomenon by chance owing to a single downsample experiment instead of multiple ones, one explanation (which we could neither proof nor reject) could be that the remaining simplex-nulliplex variants are more stable and easier to cluster.

**Figure 2:**
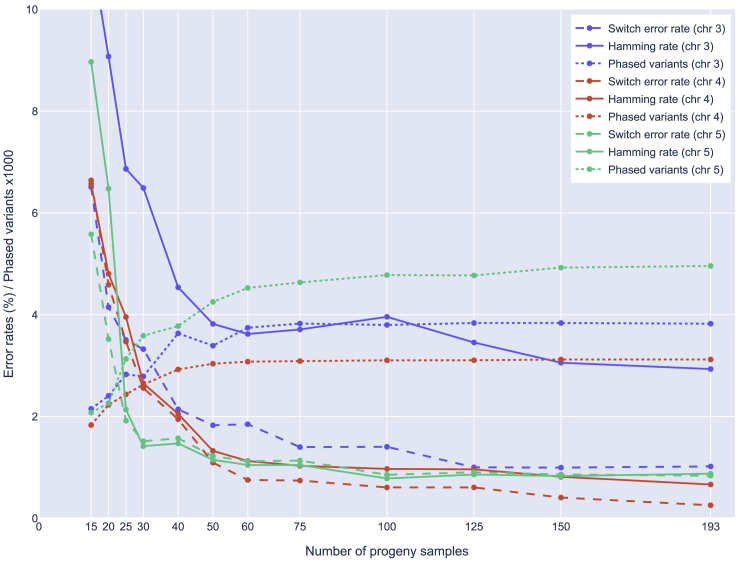
Degradation of phasing accuracy with smaller progeny pool Shows SER, HR, and number of phased variants (y axis) for different progeny pool sizes (x axis) on the three validation regions (colors). Each point represents the mean value of the 10 random samples for each of the sample sizes. Error rates are shown as percentages, variant count as thousands.

We further explored how dependent the phaser is on the number of progeny samples and parental sequencing depth. From the 193 progeny samples, we drew 10 random subsamples of sizes between 15 and 150 and reran the experiments for simplex-nulliplex variants only. The results for the three regions and three selected metrics are summarized in Figure 2. As expected, all error rates increase with smaller samples. Especially samples with less than 60 progeny begin to fall off from the rest.

### WH-PPG scales to whole chromosomes

We ran WH-PPG on all twelve chromosomes of *Solanum tuberosum* to show that it is able to scale to full genomes. We report the runtime and memory consumption, as there exists no haplotype-resolved assembly of our sample to which we can compare the resulting phasing.

**Figure 3:**
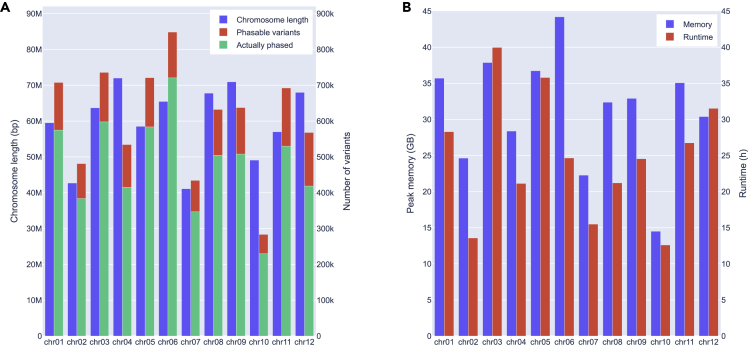
Results on whole chromosomes (A) Length and number of simplex-nulliplex variants per chromosome with smaller green bars indicating the fraction of actually phased variants. (B) Used resources per chromosome.

Figure 3 shows some statistics about the whole-chromosome runs. There is, on average, one simplex-nulliplex variant every 100 bp, of which WH-PPG phased about 80%. All chromosomes were phased separately, running on a single core each. This results in a total of 300 CPU hours for the entire genome where chromosome 3 had the highest running time (40 h) and chromosome 6 had the highest peak memory consumption (44 GB).

**Figure 4:**
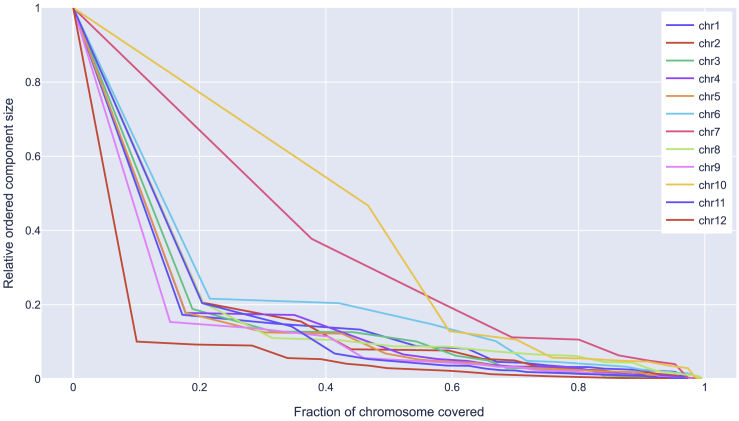
Block size estimates for different chromosome coverages The x axis represents the fraction of the chromosome covered by blocks and the y axis shows the corresponding block size (relative to the full chromosome) needed to reach the corresponding chromosome coverage.

A strength of genetic phasing is the reduced dependency on read connectivity to produce long haplotype blocks. To evaluate this, we mapped all HiFi reads against the reference genome to get an estimate of what read-based phasers could achieve with the currently available data. We removed all intervals of size at least 10 kb and with coverage less than *k* = 4 from the reference genome, as these intervals are impossible to resolve by reads alone and lead to separate phasing blocks. If we order the remaining connected components by size, we can estimate the expected block size to cover at least a fraction *x* of the genome (*x* = 0.5 to compute N50). These results are summarized for each chromosome in Figure 4. The length of the shortest block covering increasing fractions of the chromosomes diminishes quite quickly, with the N50 being less than 20% of the full chromosome length (except for chromosomes 7 and 10) and the N90 falling less than 5%.

## Discussion

The main benefit of WH-PPG is the ability to compute chromosome-scale phasings without depending on read connectivity. We showed this on a small scale using regions for which clean-looking data from another source could be used as validation. Although read-based phasing methods usually face a trade-off between accuracy and phasing block length, WH-PPG has to decide between accuracy and phasing density, because the more variant types are considered, the harder these variants become to phase. But even if large portions of heterozygous position remain unphased, we showed that the number of phasable variants remains stable over all chromosomes with an average distance of 100 bp.

With about 300X of parental coverage and almost 200 progeny samples, the amount of experimental data was relatively high. Additional tests, however, revealed that the method is still applicable to a significantly smaller subset of the data. A population size of 60 yielded sufficiently low error rates compared to the full 193 samples. We observed a noticeable decline in phased variants of about 20% after lowering the parental coverage to 25% of its original size. The accuracy of the obtained phasings did neither clearly improve nor clearly degrade after downsampling. We, therefore, conclude that a lower sequencing depth is feasible for our method, if a more sparse phasing, as a result, is acceptable for the user.

### Limitations of the study

As already pointed out in the introduction, the purpose of our method is not a complete phasing, but rather a partial phasing that is stable over long distances. This opens up the question of how the remaining variants can be inferred, such that the long-range stability is preserved. Generalizing the model to account for these variants does not seem promising as the accuracy already dropped significantly even when only incorporating duplex-nulliplex variants in our model. We rather believe that the so far unused read sequences are the key to fill in the gaps. Sufficient overlap between reads and already phased positions should allow for confident reconstructions of intermediate variants. We will implement this in a future version of WhatsHap by incorporating a partial phasing from the presented method to resolve the otherwise fragmented read clusters based on sequence overlap alone. Following this, after inferring the parental haplotypes, an even more advanced step might be to find the most likely reconstruction for each progeny based on the low-depth sequencing data. The high number of samples would then additionally allow for insights into the recombination landscape of the observed plant type.

Another open question here is how the progeny coverage influences the quality of the partial phasing or reconstruction of the progeny phases. In our experiments, we studied the effect of different population sizes using the same coverage of about 6X. For higher coverages, however, it is unknown so far whether this enables the method to run on smaller populations or whether it would yield better results overall. Without the availability of such data, the answer could only be acquired via a large simulation study.

For the phasing problem in general, it should be noted that plant genomes and the potato genome, in particular, pose more challenges that have not been addressed here. A recent study by Sun et al. (2022) revealed the extent of structural variation in the potato genome. The fact that there are large insertions or deletions on single haplotypes, let alone more complex rearrangements, and thus not always *k* haplotypes present at each site is not accounted for by current polyploid phasers, including the method described here. We identify this as an issue the community should focus on. Additional challenges are the lack of high-quality reference genomes or haplotype-resolved assemblies and thus a lack of gold-standard data to use for evaluation and the difficulty to represent the abundance of structural variation in the classic concepts of reference genomes and VCFs using a linear concept with absolute coordinates.

## STAR★Methods

### Key resources table

**Table:** 

REAGENT or RESOURCE	SOURCE	IDENTIFIER
**Deposited data**		

WGS Short read paired-end 470 bp sequencing data of parental samples “Altus” and “Colomba”	NCBI Bioproject	PRJNA718240
WGS Short read paired-end 470 bp sequencing data of progeny samples, bred from “Altus” and “Colomba”	NCBI Bioproject	PRJEB48582
Hifi CCS reads from the parental “Altus” sample	NCBI Bioproject	PRJNA778192

**Software and algorithms**		

Whatshap polyphase genetic (used version)	This paper	https://doi.org/10.5281/zenodo.6519173
Snakemake	Mölder et al., 2021	https://snakemake.github.io
GATK4	Poplin et al., 2018	https://github.com/broadinstitute/gatk;RRID:SCR_001876
PuLP	N/A	https://github.com/coin-or/pulp
Hifiasm	Cheng et al., 2021	https://github.com/chhylp123/hifiasm;RRID:SCR_021069

**Other**		

Preprocessed VCF data for three picked regions on chromosomes 3, 4 and 5 on which the phasing algorithm was run.	This paper	https://doi.org/10.5281/zenodo.6471527
Solyntus reference genome for solanum tuberosum	van Lieshout et al. (2020)	https://doi.org/10.1534/g3.120.401550

### Resource availability

#### Lead contact

Further information and requests for resources including data and code should be directed to and will be fulfilled by the lead contact, Sven Schrinner (sven.schrinner@hhu.de).

#### Materials availability

This study did not generate new unique reagents.

### Method details

Recalling the given scenario, the input of WhatsHap polyphase genetic is genotype data for two parental input samples *s*' and *s*'' and allele counts for *p* progeny samples *s*_1_, …,*s*_*p*_ and *s*' is the sample to be phased. For each heterozyguous and bi-allelic variant of *s*' we define the *majority* allele to be the more frequent of the two present alleles and the *minority* allele to be the other one. Let *m* be the number of bi-allelic variants which are heterozygous on *s*'.

In this setting, the genotype $G_{s}=G_{s}^{1}\ldots G_{s}^{m}$ of sample *s* can be expressed as a sequence of integer numbers $G_{s}^{i}\in \left\{0,\ldots ,k\right\}$ counting the occurrences of the respective minority allele among the haplotypes of *s* for each variant, where *k* is the ploidy and 1 ≤ *i* ≤ *m*. If $G_{s^{\prime }}^{i}=1$ and $G_{s^{\text{″}}}^{i}=0$, we call *i* a *simplex-nulliplex* variant. Similarly we call it a *simplex-simplex* variant if $G_{s^{\prime }}^{i}=G_{s^{\text{″}}}^{i}=1$ and a *duplex-nulliplex* variant if $G_{s^{\prime }}^{i}=2$ and $G_{s^{\text{″}}}^{i}=0$.

For each progeny sample *s* and variant *i*, let $D_{s}^{i}\left(0\right)$ and $D_{s}^{i}\left(1\right)$ be the number of occurrences of the majority and minority allele among all reads of *s*, respectively. The genotypes and allele depths form the input of our phasing algorithm.

#### Identifying and scoring phasable variants

Following the Mendelian rules, a progeny sample with even ploidy *k* inherits $\frac{k}{2}$ of its haplotypes from each of the two parents. Apart from recombination events, the two inherited haplotypes from one parent stay the same. This allows us to infer the co-occurrence of certain alleles on the parental haplotypes without directly incorporating sequencing information. Since we have to trace the origin of observed alleles among the progeny samples, only certain variant types can be phased with sufficient statistical evidence.

The easiest case is given by two simplex-nulliplex variants, where *s*' has exactly one occurrence of the minor allele on one of its haplotypes. We call these occurrences *markers* and denote the true haplotype containing this signal (i.e. minor allele) for variant *i* with *h*_*i*_. Every progeny sample either inherited both minor alleles, exactly one of them or none with different probabilities, depending on whether *h*_*i*_ = *h*_*j*_ or *h*_*i*_ ≠ *h*_*j*_ holds. Let *L*_=_(*n*_*i*_,*n*_*j*_) and *L*_≠_(*n*_*i*_,*n*_*j*_) be the probability for a progeny sample to inherit *n*_*i*_ and *n*_*j*_ minor alleles for variants *i* and *j*, given *h*_*i*_ = *h*_*j*_ and *h*_*i*_ ≠ *h*_*j*_ respectively. If there is no recombination event between *i* and *j*, then *L*_=_ and *L*_≠_ can be computed as follows:

$$\begin{array}{lllll} L_{=}\left(1,1\right)=\frac{\left(\begin{array}{l} 1\\ 1 \end{array}\right)\cdot \left(\begin{array}{l} k-1\\ k/2-1 \end{array}\right)}{\left(\begin{array}{l} k\\ k/2 \end{array}\right)} & =\frac{1}{2} & L_{\neq }\left(1,1\right) & =\frac{\left(\begin{array}{l} 2\\ 2 \end{array}\right)\cdot \left(\begin{array}{l} k-2\\ k/2-2 \end{array}\right)}{\left(\begin{array}{l} k\\ k/2 \end{array}\right)} & =\frac{\frac{k}{2}-1}{2\left(k-1\right)}\\ L_{=}\left(0,1\right)=L_{=}\left(1,0\right) & =0 & L_{\neq }\left(0,1\right)=L_{\neq }\left(1,0\right) & =\frac{\left(\begin{array}{l} 2\\ 1 \end{array}\right)\cdot \left(\begin{array}{l} k-2\\ k/2-1 \end{array}\right)}{2\cdot \left(\begin{array}{l} k\\ k/2 \end{array}\right)} & =\frac{k}{4\left(k-1\right)}\\ L_{=}\left(0,0\right)=\frac{\left(\begin{array}{l} 1\\ 0 \end{array}\right)\cdot \left(\begin{array}{l} k-1\\ k/2 \end{array}\right)}{\left(\begin{array}{l} k\\ k/2 \end{array}\right)} & =\frac{1}{2} & L_{\neq }\left(0,0\right) & =\frac{\left(\begin{array}{l} 2\\ 0 \end{array}\right)\cdot \left(\begin{array}{l} k-2\\ k/2 \end{array}\right)}{\left(\begin{array}{l} k\\ k/2 \end{array}\right)} & =\frac{\frac{k}{2}-1}{2\left(k-1\right)} \end{array}$$

**Figure 5:**
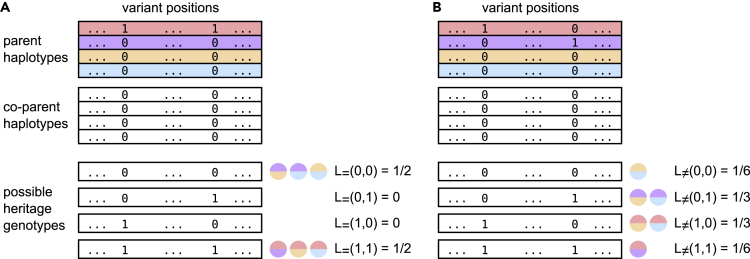
Tetraploid heritage probabilities Example for *L*_=_ and *L*_≠_ on tetraploid samples and two simplex-nulliplex variants. Each of the six possible haplotype pairs from the first parent leads to one of the four possible genotype patterns (the other parent is homozygous). (A) If both markers lie on the same haplotype, either both or no marker is inherited with the same chance. (B) If they lie on different ones, probabilities shift toward inheriting exactly one of it.

Figure 5 illustrates the probabilities for inheriting certain genotype combinations depending on allele co-occurence for tetraploid samples and two simplex-nulliplex variants.

Low coverage of the progeny samples yields uncertain genotype estimations. Therefore we rather want to explain the observed allele depths than genotypes with either assumption *h*_*i*_ = *h*_*j*_ or *h*_*i*_ ≠ *h*_*j*_. The likelihoods of allele depths $D_{s}^{i}$ and $D_{s}^{j}$ of sample *s* given *h*_*i*_ = *h*_*j*_ can be computed via (Equation 1), where all possible genotype combinations *g*_*i*_, *g*_*j*_ are assumed with a prior probability of *L*_=_(*g*_*i*_, *g*_*j*_) (for simplex-nulliplex variants all combinations with *g*_*i*_ > 1 or *g*_*j*_ > 1 have prior probability 0). The analogous case for *h*_*i*_ ≠ *h*_*j*_ uses *L*_≠_ instead of *L*_=_.

(Equation 1) $$\begin{array}{l} P\left(D_{s}^{i},D_{s}^{j}|h_{i}=h_{j}\right)=\underset{g_{i},g_{j}\in \left\{0,\ldots ,k\right\}}{\sum }P\left(D_{s}^{i},D_{s}^{j}|G_{s}^{i}=g_{i},G_{s}^{j}=g_{j}\right)\cdot L_{=}\left(g_{i},g_{j}\right)\\ \begin{array}{ll} & =\underset{g_{i},g_{j}\in \left\{0,\ldots ,k\right\}}{\sum }P\left(D_{s}^{i}|G_{s}^{i}=g_{i}\right)\cdot P\left(D_{s}^{j}|G_{s}^{j}=g_{i}\right)\cdot L_{=}\left(g_{i},g_{j}\right) \end{array} \end{array}$$

The likelihood to observe $D_{s}^{i}$ given a genotype *g*_*i*_ ∈ {0, …,*k*} follows a binomial distribution as shown in (Equation 2) where *B*_pmf_(*n*,*k*,*p*) denotes the binomial probability mass function.

(Equation 2) $$P\left(D_{s}^{i}|G_{s}^{i}=g_{i}\right)=B_{\text{pmf}}\left(\underset{\text{coverage}}{\underset{︸}{D_{s}^{i}\left(0\right)+D_{s}^{i}\left(1\right)}},D_{s}^{i}\left(1\right),\frac{g_{i}}{k}\right)$$

In principle, the calculations from above can be generalized to any pair of bi-allelic variants such that the likelihoods differ between the two cases *h*_*i*_ = *h*_*j*_ and *h*_*i*_ ≠ *h*_*j*_, respectively. However, the more complex the variants, the lower the margin between the two distributions and thus the lower the confidence for phasing decisions. Earlier studies reported a relatively low fraction of multi-allelic variants among SNPs of less than 6% (Uitdewilligen et al., 2013) and in our experiments 40% of the bi-allelic variants turned out to be simplex-nulliplex. As stated in the introduction, the phasing method aims at phasing a subset of variants for which the genetic data gives strong evidence. We will therefore restrict further descriptions to simplex-nulliplex variants only, denoted as *phasable variant pairs*.

#### Clustering variants based on Bayesian scores

In order to compute a haplotype-skeleton, we have to determine which markers of the simplex-nulliplex variants belong to which haplotype. For this purpose we chose the weighted cluster-editing model, a method to cluster nodes in a complete graph (Zahn, 1964). Each pair of nodes is assigned a real-valued score, where a positive (negative) score indicates that two nodes belong to same cluster (different clusters). The nodes correspond to simplex-nulliplex variants and the edge scores are the log-likelihood ratio of the two corresponding variants to co-occur on one haplotype versus to reside on different haplotypes. The model then asks for a minimal-cost solution with an arbitrary amount of clusters, where costs arise from node pairs that are not clustered according to the sign of their edge score. Cluster-editing is NP-hard and an exact solution is intractable for the instance sizes of our input data. Instead, we use a heuristic, which was previously described in (Böcker et al., 2011) and also used for clustering reads (instead of variants) in previous work (Schrinner et al., 2020).

For each variant pair *i*,*j* we compute the likelihoods of their signals being on the same and on different haplotypes respectively, i.e., *P*(*h*_*i*_ = *h*_*j*_) and *P*(*h*_*i*_ ≠ *h*_*j*_), given the observed allele depths among the progeny for variants *i* and *j*. The score *w*(*i*,*j*) is then defined as the logarithm over the ratio of both likelihoods as shown in Equation 3. Using Bayes’ theorem, the conditional probabilities can be expressed as likelihoods of allele depths given one of the two cases. In case of a positive (negative) score the co-occurring (disjoint) case is the more likely one, which adds a penalty to any solution where *i* and *j* end up in different (same) clusters.

(Equation 3) $$\begin{array}{l} w\left(i,j\right):=\text{log}\left(\frac{P\left(h_{i}=h_{j}|D_{s_{1}}^{i},\ldots ,D_{s_{p}}^{i},D_{s_{1}}^{j},\ldots ,D_{s_{p}}^{j}\right)}{P\left(h_{i}\neq h_{j}|D_{s_{1}}^{i},\ldots ,D_{s_{p}}^{i},D_{s_{1}}^{j},\ldots ,D_{s_{p}}^{j}\right)}\right)\\ \;=\text{log}\left(\frac{\frac{P\left(D_{s_{1}}^{i},\ldots ,D_{s_{p}}^{i},D_{s_{1}}^{j},\ldots ,D_{s_{p}}^{j}|h_{i}=h_{j}\right)\cdot P\left(h_{i}=h_{j}\right)}{P\left(D_{s_{1}}^{i},\ldots ,D_{s_{p}}^{i},D_{s_{1}}^{j},\ldots ,D_{s_{p}}^{j}\right)}}{\frac{P\left(D_{s_{1}}^{i},\ldots ,D_{s_{p}}^{i},D_{s_{1}}^{j},\ldots ,D_{s_{p}}^{j}|h_{i}\neq h_{j}\right)\cdot P\left(h_{i}\neq h_{j}\right)}{P\left(D_{s_{1}}^{i},\ldots ,D_{s_{p}}^{i},D_{s_{1}}^{j},\ldots ,D_{s_{p}}^{j}\right)}}\right)\\ \;=P\left(D_{s_{1}}^{i},\ldots ,D_{s_{p}}^{i},D_{s_{1}}^{j},\ldots ,D_{s_{p}}^{j}|h_{i}=h_{j}\right)\\ \;-P\left(D_{s_{1}}^{i},\ldots ,D_{s_{p}}^{i},D_{s_{1}}^{j},\ldots ,D_{s_{p}}^{j}|h_{i}=h_{j}\right)+\text{log}\left(\frac{1}{k-1}\right) \end{array}$$

Since the allele depths of different progeny samples are independent of each other, the likelihood of these allele depths taking certain values equals the product of the likelihood for each individual sample (see (Equations 4) and 5). These have already been resolved in (Equation 1).

(Equation 4) $$P\left(D_{s_{1}}^{i},\ldots ,D_{s_{p}}^{i},D_{s_{1}}^{j},\ldots ,D_{s_{p}}^{j}|h_{i}=h_{j}\right)=\overset{p}{\underset{l=1}{\prod }}P\left(D_{s_{l}}^{i},D_{s_{l}}^{j}|h_{i}=h_{j}\right)$$

(Equation 5) $$P\left(D_{s_{1}}^{i},\ldots ,D_{s_{p}}^{i},D_{s_{1}}^{j},\ldots ,D_{s_{p}}^{j}|h_{i}\neq h_{j}\right)=\overset{p}{\underset{l=1}{\prod }}P\left(D_{s_{l}}^{i},D_{s_{l}}^{j}|h_{i}\neq h_{j}\right)$$

A full pair-wise scoring between all phasable variants requires a quadratically growing number of computations, rendering this process intractable for chromosome-scale computations. We therefore define a *scoring window W*, which is the maximal distance between any scored pair of variants. The distance is counted in intermediate phasable variants, i.e. variant pair (*i*,*j*) will only be scored if *i*−*W* ≤ *j* ≤ *i*+*W*.

With recombination events the computation of *L*_=_ and *L*_≠_ becomes more involved as it would require knowledge about (possibly local) recombination rates. Even though we limit the distance between scored variant pairs, there is a small chance for each of the progeny samples to introduce some noise to the score due to a recombination event. Since there are usually only a few recombination events present on each chromosome and considering that the scoring window spans less than 1% of the chromosome in our experiments, only a small minority of progeny samples would be affected for each computation. We therefore decided to leave this source of noise in the model in favor of not introducing any assumptions about recombination densities.

While a large scoring-window *W* reduces the risk of switch errors due to locally (but not globally) optimal clustering it also increases computation time for scoring and clustering. As a compromise we used a sparse scoring pattern which only scores every sixth possible variant pair on average: For every variant *i* take the $\left\lceil \frac{W}{24}\right\rceil$ consecutive variants after *i* first. From there on, select every third variant until $\left\lceil \frac{W}{12}\right\rceil$ variants are selected. Proceed with every seventh variant for the next $\left\lceil \frac{W}{24}\right\rceil$ variants and then select every 13th variant until the bounds of the window are reached. Choosing *W* = 1,500 and thus having 250 scoring partners for each variant in both directions proved to be a good compromise between speed and accuracy. All unscored variant pairs are assumed to have a score of 0 and can be ignored in the model.

#### Assigning haplotypes: interval scheduling

Cluster editing does not necessarily yield exactly *k* clusters, which would directly result in a phasing of all phasable variant pairs. In practice the number of clusters is much higher with many small and even singleton clusters due to different sorts of errors in the data. There are two ways to deal with this issue: We could either find an assignment for all clusters to the *k* haplotypes, such that the contradiction to the scores is minimized or we could find a maximum conflict-free subset of clusters which explains the highest possible number of variants. Since we already limited the model to simplex-nulliplex variants for the sake of accuracy over completeness, it appears more logical to follow the latter approach.

Let *C*: = {*c*_1_,…*c*_*n*_} be the set of computed clusters from the previous step and let min(*c*_*i*_) and max(*c*_*i*_) be the lowest and highest index for all variant indices in *c*_*i*_ for 1 ≤ *i* ≤ *n*, respectively. If two clusters *c*_*i*_,*c*_*j*_ do not overlap and there is at least a full scoring-window of *W* variant positions in between them, i.e. either max(*c*_*i*_)+*W* ≥ min(*c*_*j*_) or max(*c*_*j*_)+*W* ≥ min(*c*_*i*_) holds, these clusters are compatible and can be assigned to the same haplotype. Thus, the goal is to find an assignment of each cluster to one of the *k* haplotypes or to remain unphased, such that the sum of contained variants *w*_*i*_ for each phased cluster *c*_*i*_ is maximized.

In scheduling theory, this problem is known as weighted interval scheduling on *k* identical machines. Each cluster *c*_*i*_ corresponds to a job with fixed start time min(*c*_*i*_), fixed end time max(*c*_*i*_) and profit *w*_*i*_. In (Arkin and Silverberg, 1987), the authors both gave a formulation as Integer Linear Program (ILP) and as a minimum cost flow. They point out that the matrix of the constraint coefficients is unimodular, such that the ILP is solvable in polynomial time.

Here, we use an alternative and easy to implement ILP formulation, which can still be solved efficiently in practice. It contains a set of binary variables $x_{i}^{j}$ for 1 ≤ *i* ≤ *n*,1 ≤ *j* ≤ *k* where *n* is the number of clusters. If cluster *i* is assigned to haplotype *j*, $x_{i}^{j}$ is set to 1 and to 0 otherwise. Let *X*:={(*i*,*l*)|*c*_*i*_ incompatible to *c*_*l*_}. Then an optimal cluster assignment is found by solving the following ILP:

(Equation 6) $$\text{max}\;\overset{n}{\underset{i=1}{\sum }}\overset{k}{\underset{j=1}{\sum }}x_{i}^{j}w_{i}$$

(Equation 7) $$\text{subject to}\;\;x_{i}^{j}+x_{l}^{j}\;\leq \;1\;\forall \;1\leq \;j\;\leq k,\;\forall \;\left(i,l\right)\;\in \;X$$

(Equation 8) $$\overset{k}{\underset{j=1}{\sum }}x_{i}^{j}\;\leq \;1\;\forall \;1\leq \;i\;\leq \;n$$

(Equation 9) $$x_{i}^{j}\;\in \;\left\{0,1\right\}\;\forall \;1\;\leq \;i\;\leq \;n,\;\forall 1\;\leq \;j\;\leq \;k$$

#### Implementation details

The scheduling ILP is implemented in PuLP using the free solver CBC. Experiments have been organized as pipeline via Snakemake (Mölder et al., 2021). All tests were run on an AMD Epyc 7742 processor with 64 cores and 1TB of memory running on Debian.

## Data Availability

• There are three separate sequencing data sets: Short read data for two parental samples, short read data for the progeny samples and Hifi sequencing data for the parental samples. All three data sets have been uploaded on NCBI database as SRA. Accession numbers are listed in the key resources table. • All algorithms are implemented as part of the WhatsHap phasing suite (Patterson et al., 2015). The status of all original code by the time the experiments were run has been deposited at Zenodo and is publicly available as of the date of publication. Instructions how to run this code have been included. Existing software, which is either directly used by the phasing algorithm or contributed significantly to the data procession are Hifiasm, Snakemake and PuLP. A reference to these tools is listed in the key resources table. • Any additional information required to reanalyze the data reported in this paper is available from the lead contact upon request. Except for read mapping and initial variant calling, all experiments have been run through a snakemake pipeline, which has been deposited at Zenodo. In order to enable access to the input VCF files for the novel phasing algorithm, we separately uploaded this processed intermediary files under a separate DOI on Zenodo, which is listed in the key resources table.
